# The P2Y_6_ Receptor Mediates *Clostridium difficile* Toxin-Induced CXCL8/IL-8 Production and Intestinal Epithelial Barrier Dysfunction

**DOI:** 10.1371/journal.pone.0081491

**Published:** 2013-11-22

**Authors:** Ashleigh Hansen, Laurie Alston, Sarah E. Tulk, L. Patrick Schenck, Michael E. Grassie, Basmah F. Alhassan, Arun Teja Veermalla, Samir Al-Bashir, Fernand-Pierre Gendron, Christophe Altier, Justin A. MacDonald, Paul L. Beck, Simon A. Hirota

**Affiliations:** 1 Department of Physiology & Pharmacology, University of Calgary, Calgary, Alberta, Canada; 2 Department of Microbiology, Immunology & Infectious Disease, University of Calgary, Calgary, Alberta, Canada; 3 Department of Biochemistry & Molecular Biology, University of Calgary, Calgary, Alberta, Canada; 4 Department of Medicine, University of Calgary, Calgary, Alberta, Canada; 5 Department of Pathology and Laboratory Medicine, Jordan University of Science and Technology, Irbid, Jordan; 6 Department of Anatomy & Cell Biology, University of Sherbrooke, Sherbrooke, Quebec, Canada; Institute Pasteur, France

## Abstract

*C. difficile* is a Gram-positive spore-forming anaerobic bacterium that is the leading cause of nosocomial diarrhea in the developed world. The pathogenesis of *C. difficile* infections (CDI) is driven by toxin A (TcdA) and toxin B (TcdB), secreted factors that trigger the release of inflammatory mediators and contribute to disruption of the intestinal epithelial barrier. Neutrophils play a key role in the inflammatory response and the induction of pseudomembranous colitis in CDI. TcdA and TcdB alter cytoskeletal signaling and trigger the release of CXCL8/IL-8, a potent neutrophil chemoattractant, from intestinal epithelial cells; however, little is known about the surface receptor(s) that mediate these events. In the current study, we sought to assess whether toxin-induced CXCL8/IL-8 release and barrier dysfunction are driven by the activation of the P2Y_6_ receptor following the release of UDP, a danger signal, from intoxicated Caco-2 cells. Caco-2 cells express a functional P2Y_6_ receptor and release measurable amounts of UDP upon exposure to TcdA/B. Toxin-induced CXCL8/IL-8 production and release were attenuated in the presence of a selective P2Y_6_ inhibitor (MRS2578). This was associated with inhibition of TcdA/B-induced activation of NFκB. Blockade of the P2Y_6_ receptor also attenuated toxin-induced barrier dysfunction in polarized Caco-2 cells. Lastly, pretreating mice with the P2Y_6_ receptor antagonists (MSR2578) attenuated TcdA/B-induced inflammation and intestinal permeability in an intrarectal toxin exposure model. Taken together these data outline a novel role for the P2Y_6_ receptor in the induction of CXCL8/IL-8 production and barrier dysfunction in response to *C. difficile* toxin exposure and may provide a new therapeutic target for the treatment of CDI.

## Introduction

*Clostridium difficile* (*C. difficile*), a Gram-positive spore-forming anaerobic bacterium, is a leading cause of nosocomial diarrhea worldwide. Hospital outbreaks, the increased occurrence of community-acquired infections and the growing threat of antibiotic resistance highlight the need for new therapeutics to treat *C. difficile* infections (CDI) [1-3].

Through the release of two large toxins, toxin A (TcdA) and toxin B (TcdB), *C. difficile* triggers intestinal tissue damage and a robust inflammatory response resulting in relapsing diarrhea, pseudomembranous colitis, toxic megacolon and, in severe cases, death [4]. TcdA and TcdB are glucosyltransferases that glucosylate and inhibit monomeric G-proteins, such as Cdc42, Rho and Rac1, leading to changes in cytoskeletal function, cell rounding and the loss of intestinal epithelial barrier function [5]. In addition to damaging the intestinal epithelial layer, TcdA and TcdB trigger the release of inflammatory mediators from intestinal epithelial cells (IECs) and monocytes/macrophages [6-8]. Toxin-induced CXCL8/IL-8 release from IECs is well documented and thought to play a key role in the recruitment of inflammatory cells into intestinal tissue [9]. 

One of the hallmarks of CDI is the massive influx of neutrophils into the colonic mucosa [4]. This inflammatory response may play a role in controlling the severity of CDI, but may also contribute to its pathogenesis. Animal studies have indicated that the neutrophilic response is required to resolve CDI in the absence of a therapeutic intervention [10-12]. Notably, Hasegawa et al. (2011) reported that Nod1-/- mice displayed more severe CDI, an observation linked to inadequate neutrophil recruitment and increased systemic bacterial translocation [10]. On the other hand, the influx of neutrophils may contribute to the tissue damage observed in CDI [13]. Studies targeting the early inflammatory responses triggered by TcdA and TcdB have reported reduced disease severity [14-17]. Indeed strategies to neutralize TcdA and TcdB also proven effective in reducing CDI severity [18-20]. We have previously reported that inhibiting the toxin-induced production of IL-1β, and the subsequent immune cell infiltration, protected mice from toxin-induced intestinal tissue damage [14,21]. Clinical studies have observed a strong correlation between elevated cytokine production and the severity CDI, a correlation that holds true even after the toxin burden was taken into account [13]. These data suggest that an exaggerated immune response may contribute to the pathogenesis of CDI. 

In IECs, *C. difficile* toxins trigger cell stress [22] and induce cell death through apoptosis [22-25] and necrosis [26,27]. In many cases, stressed or dying cells release a variety of endogenous mediators, such as ATP, UDP and HMGB1, that can activate receptors on neighboring cells [28,29]. These substances, termed “danger signals”, are thought to initiate cellular events that help the rid the system of the offending agent or enhance the removal of dead cell material. Extracellular nucleotides, such as UDP, have been characterized as danger signals in a number of different systems and trigger the production of inflammatory mediators, such as CXCL8/IL-8 [30,31] and increase the ability of macrophages to bind and phagocytose apoptotic bodies [27]. In the context of the gastrointestinal tract, inflammatory stress has been reported to trigger the release of nucleotides, such as UDP, that can initiate tissue inflammation and following the production of CXCL8/IL-8 through the activation of the P2Y_6_ receptor [31,32]. 

The P2Y_6_ receptor is a G-protein coupled receptor that signals via Gq/11-dependent pathways that include IP_3_-dependent mobilization of intracellular calcium stores, stimulation of protein kinase C and induction of Rho-associated kinase (ROCK) signaling through p63RhoGEF [33], the latter of which can modulate cell-cell contacts triggering barrier dysfunction in endothelial [34] and epithelial cells [35,36]. In the context of inflammation, it has been demonstrated that P2Y_6_ activation can activate NFκB signaling in a number of cell types [37,38], although the exact signaling events that drive this process have yet to be fully elucidated. 

In the present study, we hypothesized that TcdA/B-induced CXCL8/IL-8 release and intestinal barrier dysfunction involves the action of extracellular UDP released from stressed or dying cells resulting in the autocrine/paracrine activation of the P2Y_6_ receptor. Herein we report that Caco-2 cells express a functional P2Y_6_ receptor and release detectable levels of UDP when exposed to TcdA/B. TcdA/B-induced CXCL8/IL-8 production and release from Caco-2 cells was blocked by the selective P2Y_6_ receptor antagonist MRS 2578. Furthermore, intestinal epithelial barrier function was protected when Caco-2 cells were pretreated with MRS 2578. Lastly, using a mouse model of toxin-induced inflammation and tissue damage, we report that P2Y_6_ receptor blockade/inhibition attenuates TcdA/B-induced colonic inflammation and intestinal epithelial barrier dysfunction. 

## Methods

### Toxin preparation

*C. difficile* TcdA/B was produced as described previously [21,39]. Briefly, *C. difficile* strain (VPI - ATCC 43255, designation VPI 10463) was grown in brain-heart infusion (BHI) media under anaerobic conditions. Dialysis tubing containing sterile phosphate-buffered saline (PBS) was inoculated with an overnight culture and suspended in 1000 mL of BHI media. Cultures were harvested at day 5 post-inoculation, centrifuged, (10,000 x *g*, 60 min), passed through a 0.22 μm filter to remove bacterial spores and cells and then concentrated via centrifugation through a 100-kDa cut-off spin filter (Chemicon, Millipore, Billerica, MA). This preparation was used as the TcdA/B mixture for most experiments. In some experiments, toxin A (TcdA) and toxin B (TcdB), were further purified from TcdA/B mixture and assess for purity by SDS-PAGE and western blotting as published previously [21,39]. 

### Cell culture

Caco-2 cells (ATCC - HTB-37; Organism: Homo sapiens, human / Tissue: Colon / Cell Type: Epithelial cells) were seeded at a density of 5 x 10^7^ cells/mL onto 12-well plates and allowed to grow for 7-days post-confluence in Dulbecco's modified Eagle medium supplemented with 20% fetal bovine serum and penicillin-streptomycin (100 μg/mL, 1 nmol/L; Invitrogen, Carlsbad, CA). All experiments were performed at day 7 post-confluence. For permeability assays, 5 x 10^4^ Caco-2 cells were plated on Costar 12-well polystyrene permeable inserts (Corning, Tewksbury, MA) and were grown for 14-days post-confluence to allow for polarization.

### Calcium imaging in Caco-2 cells

Calcium signaling was monitored in Caco-2 cells loaded with Fluo-3 AM (1 μM for 20 min; Life Technologies/Invitrogen, Carlsbad, CA).  Briefly, cells were perfused (approximately 2 mL/min.) with NaCl-based extracellular solution containing (in mM): NaCl, 130; KCl, 3; MgCl_2_, 0.6; CaCl_2_, 2; NaHCO_3_, 1; HEPES, 10; glucose, 5) at room temperature for 10 min. Band limited excitation (420–495 nm) was provided by a fluorescence light source system (Olympus, Richmond Hill, Canada).  Cells were imaged using an Olympus IX51 microscope with the digital imaging software (Olympus, Richmond Hill, Canada). Images were acquired using a CCD camera (Olympus, Richmond Hill, Canada) at an effective sampling rate of 1 Hz, and digitized using DV video format [40]. Regions of interest (ROIs) were fitted around the perimeter of cells using ImageJ software and intensity variations for each ROI were corrected for background levels and expressed in relation to a baseline fluorescence level preceding P2Y_6_ stimulation with 5-OMe-UDP to obtain ΔF/F fluorescent intensity values.

### UDP quantification

Caco-2 cells were cultured in phenol-free Dulbecco's modified Eagle medium supplemented with 20% fetal bovine serum and penicillin-streptomycin (100 μg/mL, 1 nmol/L; Invitrogen, Carlsbad, CA). Following a 16-hr treatment with TcdA/B, culture supernatants were sterile filtered and UDP detected by HPLC as described previously by Grbic et al. (2008) [31]. Control supernatants were spiked with UDP (100 μM) or TcdA/B (10 μg/mL) post-extraction, acting as a comparative standard for elution time and peak quantification. 

### CXCL8/IL-8 Quantification

Confluent Caco-2 monolayers were treated with TcdA/B or 5-OMe-UDP and various pharmacological blockers (P2Y_6_ receptor antagonist MRS2578 – 1 and 10 μM, non-selective P2 receptor antagonist PPADS – 10 and 100μM) for 16 hrs. Following the treatment period, culture supernatants were removed, centrifuged to remove non-adherent cells (2000 x g, 10 minutes at 4°C) and then flash frozen for subsequent analysis. CXCL8/IL-8 was quantified by ELISA (R&D Systems Inc. Minneapolis, MN), according to the manufacturer’s instructions. 

### Quantitative real-time PCR

Caco-2 monolayers were treated and collected in Trizol at various time-points. RNA was isolated according to manufacturer's protocol (Invitrogen, Carlsbad, CA). Total RNA was reverse transcribed with the RT^2^ First-strand Kit (SABiosciences, Frederick, MD). CXCL8/IL-8 transcript expression was assessed using an ABI 7500 real-time PCR thermocycler. PCR reactions were composed of validated primers from SABiosciences, cDNA and RT2 real-time SYBR Green/Rox PCR master mix (SABiosciences; CXCL8/IL-8 – Cat. #PPH00568A; Refseq Accession #: NM_000584; b-actin – Cat. #PPH00073G; Refseq Accession #: NM_001101). Amplification plots were examined with the accompanying Sequence Detection Software to determine the threshold cycle (Ct). In all reactions endogenous control (β-actin; ACTB) was amplified, and the Ct was determined. Data are expressed a fold-change calculated using the ΔΔCt method [41]. 

### Assessment of cell viability

Cell viability was quantified by measuring the release of lactate dehydrogenase using the CytoTox 96 Non-Radioactive Cytotoxicity Assay (LDH assay; Promega, Madison, WI) performed according to the manufacturer's instructions.

### Assessment of toxin activity

The glucosyltransferase activity of the TcdA/B mixture was assessed by western blotting lysates isolated from TcdA/B-treated Caco-2 cells for Rac1 using an anti-Rac1 antibody the that detects the non-glucosylated form (Mab 102, BD Biosciences, Mississauga, Canada) as described previously by Genth et al. (2006) [42]. 

### In vitro permeability assay

Permeability assays were performed as previously described [43]. Briefly, Caco-2 cells plated on Costar 12-well polystyrene permeable inserts (Corning, Tewksbury, MA) were given fresh medium every other day and grown 14-days post-confluence to allow for polarization. To assess changes in permeability, fluorescein isothiocyanate-dextran (FITC-dextran, molecular weight 4 kDa, 0.5 mg/mL, Sigma, Oakville, Canada) was added to the apical compartment of the plate and medium samples from the basolateral compartment of the plate at various time-points following the initiation of the experiment. Cells were treated with 5-OMe-UDP (dissolved in sterile ddH_2_O, Tocris Bioscience, Burlington, Canada) and TcdA/B in the presence and absence of MRS2578 (dissolved in sterile DMSO, Sigma, Oakville, Canada) and media sampled from the basolateral compartment at 0, 2 and 4 hr post-stimulation. The movement of FITC-dextran from the apical to the basolateral compartment was assessed by reading the sampled media on a fluorometric plate reader. Data are expressed as arbitrary fluorescent units. 

### ZO-1 immunofluorescence staining

Caco-2 cells were plated at 1.2 x 10^5^ cells/mL on 8-well chamber slides (Lab-Tek® Chamber Slide, Thermo Fisher Scientific Inc., Toronto, Canada). Cells were grown for 7-days post-confluence and then used for experiments. Cells were treated with 5-OMe-UDP (dissolved in sterile ddH_2_O; Tocris Bioscience, Burlington, Canada) and TcdA/B in the presence and absence of MRS2578 for 4 hr. At the end of the experiment the cells were stained as described previously [43]. Briefly, cells were rinsed twice with ice-cold PBS and then fixed with ice-cold methanol for 30 min at 4 °C. Following fixation, the cells were blocked with normal donkey serum (15 min at room temperature) and then incubated with rabbit anti-ZO-1 primary antibody (1:100 dilution, 1 hr at 37 °C; Invitrogen, Carlsbad, CA). Cells were then rinsed with PBS twice and incubated with Cy5-conjugated secondary goat anti-rabbit IgG (1:500 dilution, 1 hr at 37 °C; Jackson ImmunoResearch, West Grove, PA). Cells were then rinsed 3 times with PBS and coverslips affixed using Fluorosave Reagent; (Calbiochem, Billerica, MA). Cells were viewed by fluorescence microscopy, and images were captured with a digital DS-Fi1 camera (Nikon, Mississauga, Canada). 

### Intrarectal instillation of *C. difficile* toxins

All mice used in our studies were male between 8 and 10 weeks of age. C57/Bl6 (Charles River, St. Constant, QC, Canada). The instillation of *C. difficile* TcdA/B was performed as described previously [21]. Briefly, a 5F infant feeding tube catheter with side ports (Mallinckrodt Inc., St. Louis, MO; catalogue No. 85771) was lubricated with water-soluble personal lubricant (Healthcare Lubricating Jelly, Toronto, ON, Canada) and inserted 2.5 cm (measured from the midway point between the 2 catheter side ports) into the colon. At this point 100 μL of solution was slowly administered while pressure was applied to the anal area to prevent leakage. Following the injection of the solution, the tube was slowly removed and the rectal pressure was maintained for 30 seconds. *C. difficile* TcdA/B (50 μg) was diluted in PBS to allow for a uniform 100 μL of solution to be injected. Control animals were treated with 100 μL of PBS. Mice were euthanized 4 hr post-instillation and tissue and plasma isolated for experimental outcomes. All animal experiments were approved by the Health Sciences Animal Care Committee of the University of Calgary and conform to the guidelines set forth by the Canadian Council for Animal Care.

### Tissue Myeloperoxidase Assay

Tissue myeloperoxidase (MPO) activity was determined as described previously [21,39]. MPO activity was measured in units per milligram of tissue, where 1 unit of MPO was defined as the amount needed to degrade 1 μmol of H_2_O_2_ per min at room temperature.

### *In vivo* Intestinal Permeability Assay

To assess intestinal permeability we measured the movement of FITC-dextran (molecular weight 4 kDa; Sigma-Aldrich, Oakville, Ontario, Canada) from the lumen of the gastrointestinal tract into the plasma as described previously [21]. All mice were administered FITC-dextran (60 mg/100 g body weight) by oral gavage 1 hr prior to the treatment with TcdA/B or PBS vehicle control. After the 4 hr exposure period whole blood was obtained by cardiac puncture at the time of euthanasia. Plasma was isolated and FITC-dextran measurements were performed in triplicate in a fluorometric plate reader at 488 nm. Data are expressed as total fluorescence units in 100 μL of plasma.

### Histological Assessment

The severity of TcdA/B-induced colitis was scored histologically using two different parameters on coded, hematoxylin and eosin stained slides in a blinded fashion by a board-certified pathologist. An inflammation score was used to assess the severity of the inflammatory response: 0, normal; 1, increased number of inflammatory cells in lamina propria; 2, increased number of inflammatory cells in submucosa; 3, dense inflammatory cell mass, but not transmural in nature; 4, transmural inflammation. Second, an assessment of each section was performed to determine an estimate of the percentage of the colonic tissue section exhibiting architectural changes (% architecture change).

### Statistical Analysis

Data are expressed as mean +/- the standard error of the mean. The Student’s t-test was used for single comparisons of parametric data. One-way ANOVA tests were used for multiple comparison analyses on parametric data followed by Tukey’s Multiple Comparison Test to determine statistical differences. Mann-Whitney tests were used to assess single non-parametric comparisons. 

## Results

### Caco-2 cells express the functional components of the P2Y_6_ signaling system

**Figure 1:**
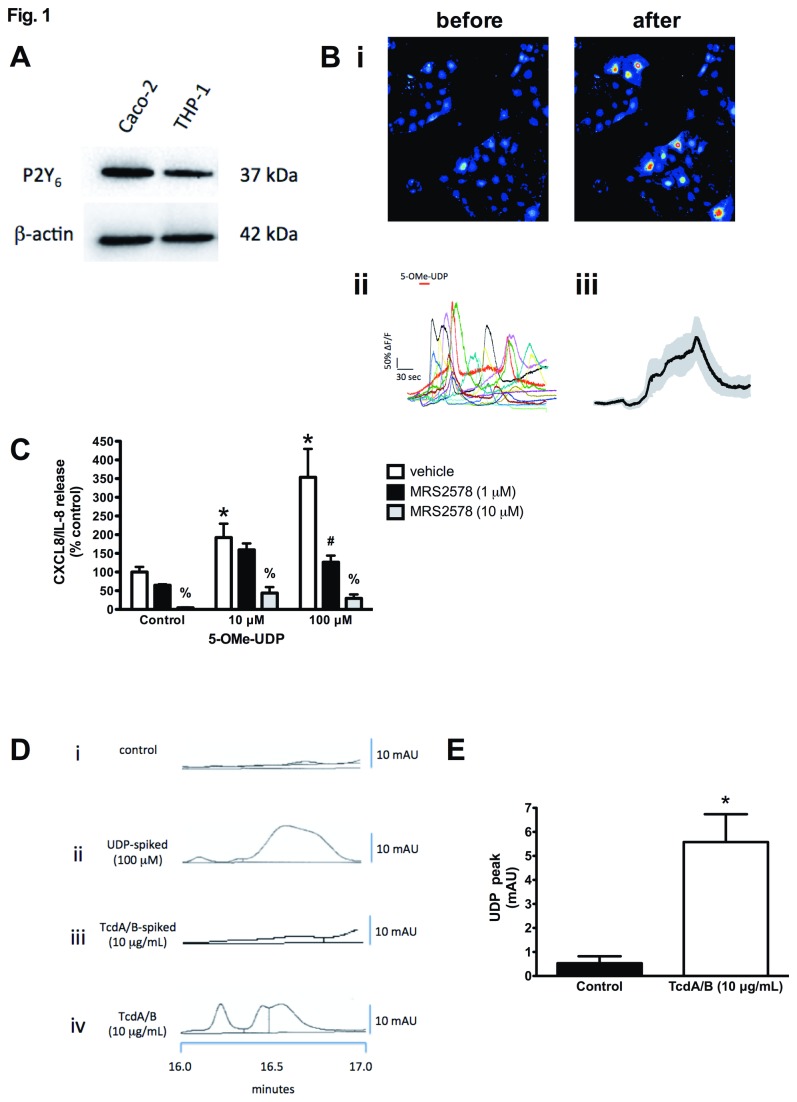
*C. difficile* TcdA/B triggers the release of UDP from Caco-2 cells that express a functional P2Y_6_ receptor. (A) Western blot analysis of lysates reveals the expression of the P2Y_6_ receptor in differentiated Caco-2 cells and PMA-differentiated THP-1 macrophages (included as positive control). (B) Stimulation of the Caco-2 cells with the selective P2Y_6_ receptor agonist 5-OMe-UDP (1 μM) increases intracellular calcium concentrations as assessed by fluorescence imaging. (B-i) Pseudocolour images of Caco-2 cells before and after 5-OMe-UDP treatment. (B-ii) Representative traces of individual cells challenged with 5-OMe-UDP. (B-iii) The mean of the 5-OMe-UDP-induced calcium responses (n=46; grey denotes the standard error of the mean). (C) P2Y_6_ receptor agonist 5-OMe-UDP triggers CXCL8/IL-8 release from Caco-2 cells, an effect that blocked by the potent P2Y_6_ receptor antagonist MRS2578. N = 6; * denotes p<0.05 compared to control; # denotes p<0.05 compared to vehicle; % denotes p<0.05 compared to vehicle and 1 μM MRS 2578. (D) TcdA/B triggers the release of UDP as assessed by HPLC. i – control treated culture supernatant; ii – UDP-spiked control culture supernatant (100 μM UDP); iii – TcdA/B-spiked control culture supernatant (10 μg/mL); iv – TcdA/B-treated cell culture supernatant (10 μg/mL; 16 hr). (E) Summary data from HPLC measurement of TcdA/B-induced UDP release. N=5; * denotes p<0.05.

The release of CXCL8/IL-8 from TcdA/B treated IECs has been well documented, although the mechanism(s) through which this occurs have yet to be fully elucidated [8,44-46]. We hypothesized that this process involves the release of UDP and autocrine/paracrine activation of the P2Y_6_ receptor and downstream induction of NFκB-dependent CXCL8/IL-8 transcription. To test this hypothesis we first examined if Caco-2 cells express a functional P2Y_6_ receptor. Indeed, confluent Caco-2 monolayers express the P2Y_6_ receptor as assessed by western blotting (Figure 1A). Using calcium imaging assays on Caco-2 cells, application of 5-OMe-UDP, a potent and selective P2Y_6_ receptor agonist, evoked an increase in calcium signals (Figure 1B; i – pseudocolour images of Caco-2 cells before and after stimulation with 5-OMe-UDP; ii – representative traces of 5-OMe-UDP-induced calcium responses; iii – the mean calcium response triggered by 5-OMe-UDP). Taken together these data suggest the expression of a functional P2Y_6_ receptor in Caco-2 cells. To examine whether P2Y_6_ receptor activation could trigger the production of CXCL8/IL-8, an observation published previously [31,32], we stimulated Caco-2 cells with 5-OMe-UDP for 16 hr and measured CXCL8/IL-8 release by ELISA. 5-OMe-UDP triggered a dose-dependent increase in CXCL8/IL-8 release, which was completely blocked by pretreating the cells with MRS2578 (10 μM; Figure 1C), a selective inhibitor of the P2Y_6_ receptor [47]. We next sought to determine whether exposing Caco-2 cells to TcdA/B could trigger the release of UDP, an endogenous ligand for the P2Y_6_ receptor. Indeed, TcdA/B induced significant UDP release as assessed by HPLC. Spiking control culture supernatants with UDP (100 μM) post-isolation revealed a unique deflection between 16 and 17 minutes of elution (Figure 1D, trace ii). Isolated culture supernatants from TcdA/B-treated cells exhibited a similar deflection as the UDP-spiked control supernatants between 16 and 17 minutes (Figure 1D, trace iv); a deflection not observed in control (Figure 1D, trace i) or TcdA/B-spiked supernatants (Figure 1D, trace iii). The magnitude of the TcdA/B-induced deflection was quantified and revealed a significant increase in UDP release following the 16-hr treatment period (Figure 1E). 

### TcdA/B-triggers CXCL8/IL-8 release through a P2Y_6_-mediated pathway

**Figure 2:**
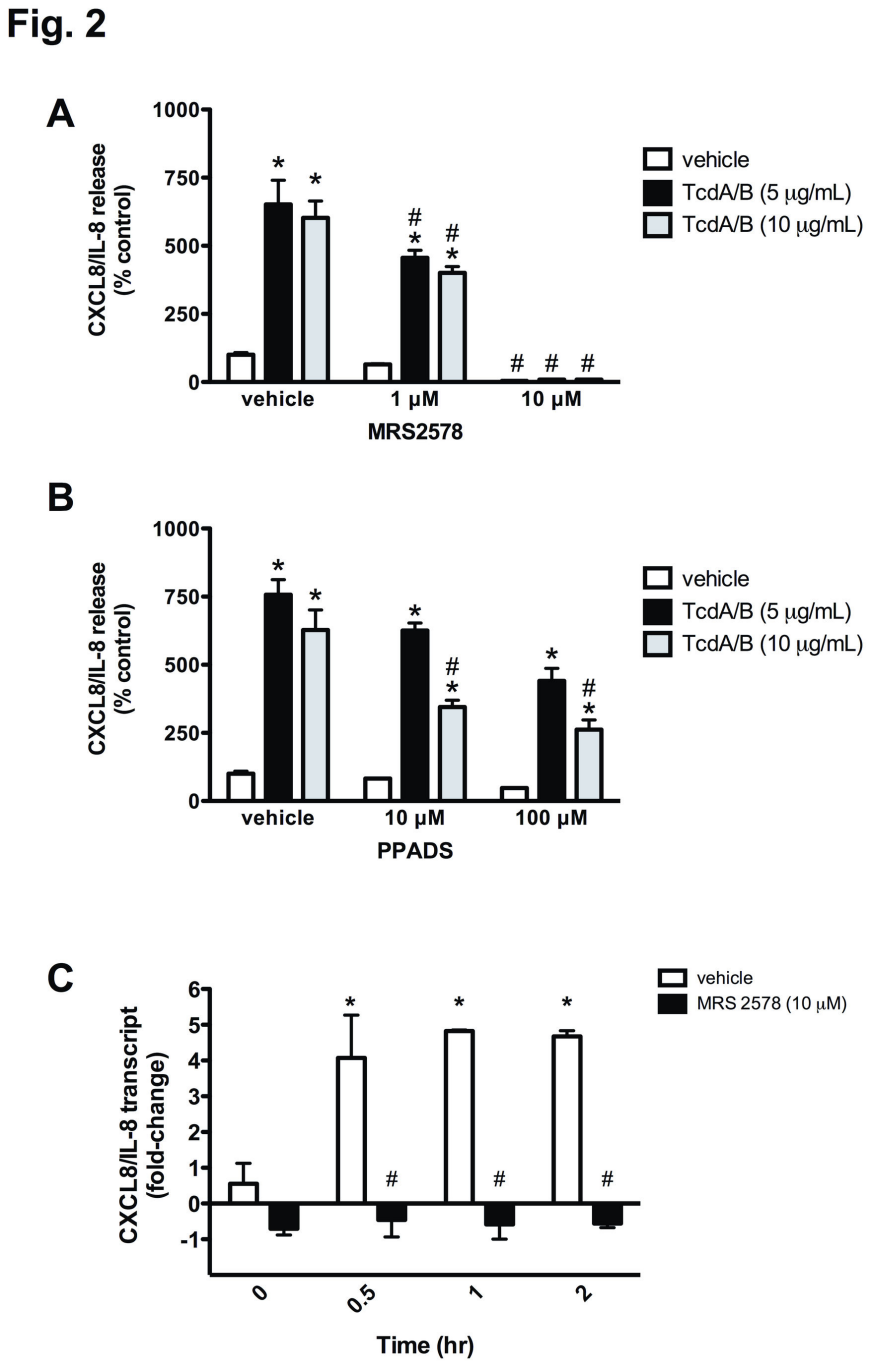
*C. difficile* TcdA/B triggers the production and release of CXCL8/IL-8 through the activation of the P2Y_6_ receptor. (A) TcdA/B-induced CXCL8/IL-8 release was significantly reduced by the selective P2Y_6_ receptor antagonist MRS2578. N = 5; * denotes p<0.05 compared to vehicle (BHI culture broth). # denotes p<0.05 compared to vehicle-treated TcdA/B-stimulated cells. (B) TcdA/B-induced CXCL8/IL-8 release is sensitive to the selective P2-receptor antagonist PPADS. N = 5; * denotes p<0.05 compared to vehicle (BHI culture broth). # denotes p<0.05 compared to vehicle-treated TcdA/B stimulated cells. (C) Pharmacological antagonist of the P2Y_6_ receptor attenuates TcdA/B-induced CXCL8/IL-8 transcription in Caco-2 IECs. N = 5; * denotes p<0.05 compared to time 0. # denotes p<0.05 compared to vehicle-treated TcdA/B stimulated cells (10 μg/mL).

Given that Caco-2 cells express a functional P2Y_6_ signaling system and the TcdA/B exposure triggered UDP release, we next sought to determine if TcdA/B-induced CXCL8/IL-8 release was mediated through activation of the P2Y_6_ receptor. Inhibiting the P2Y_6_ receptor by pretreatment of Caco-2 cells with MRS2578 (1 μM and 10 μM) significantly reduced TcdA/B-induced CXCL8/IL-8 release (Figure 2A). Furthermore, the less potent P2-receptor antagonist PPADS (10 μM and 100 μM) reduced TcdA/B-induced CXCL8/IL-8 release to a lesser extent (Figure 2B). Since inhibition of the P2Y_6_ receptor with MRS2578 completely abolished CXCL8/IL-8 production, we next assessed TcdA/B-induced CXCL8/IL-8 transcription at early time-points post-receptor stimulation. The production of CXCL8/IL-8 transcript in response to TcdA/B treatment could be observed as early as 30 min following stimulation and continued to remain elevated up to 2 hr post-TcdA/B stimulation (Figure 2C). Inhibiting the P2Y_6_ receptor with MRS2578 (10 μM) completely abolished TcdA/B-induced CXCL8/IL-8 transcription (Figure 2C). Taken together these data suggest that the P2Y_6_ receptor is involved in the TcdA/B-induced CXCL8/IL-8 response in IECs through the induction of gene transcription. 

**Figure 3:**
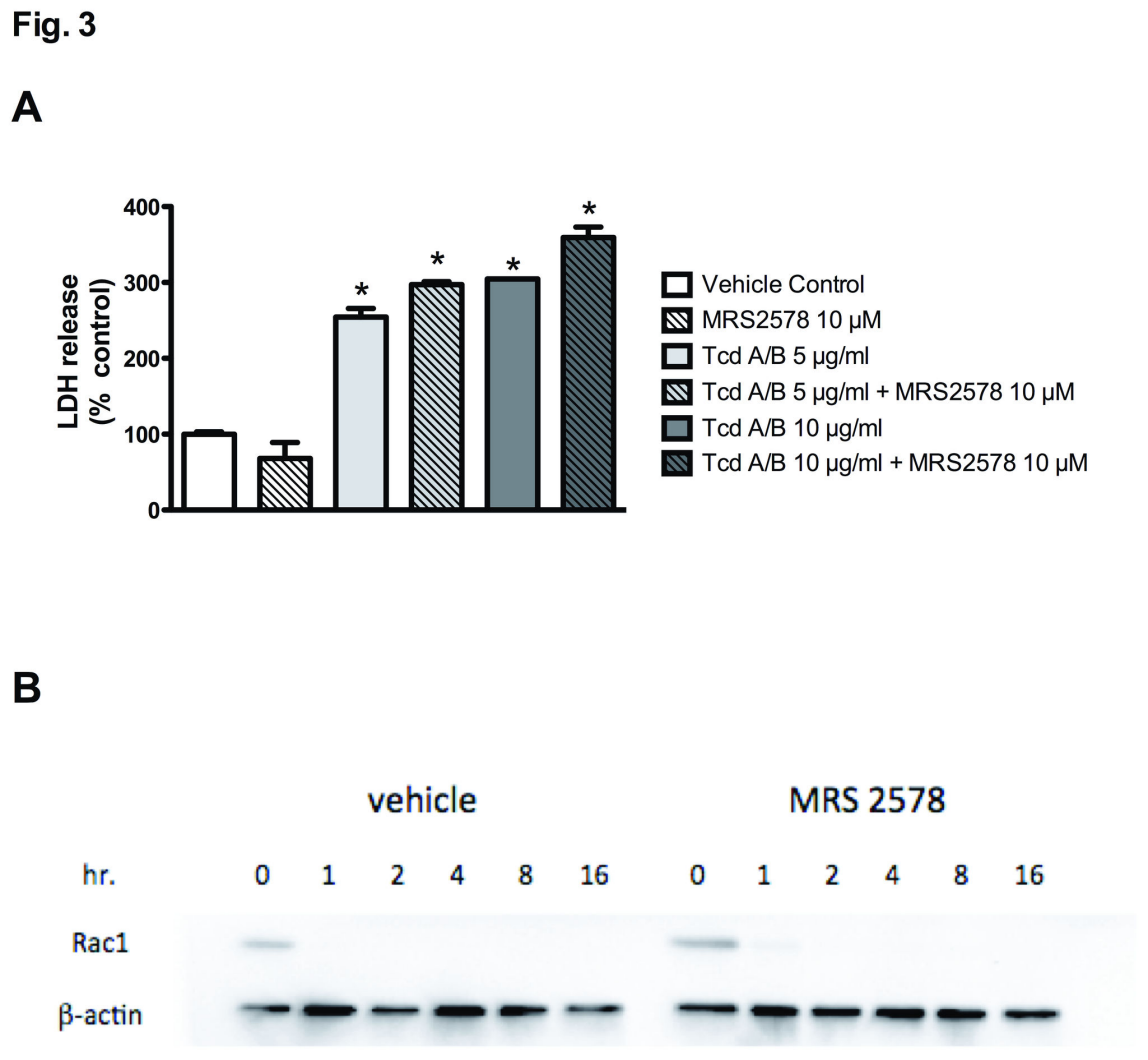
TcdA/B-induced cell death and Rac1 modification are not affected by MRS2578. (A) TcdA/B-induced cell death, as assessed by lactate dehydrogenase release (LDH), is not affected by the selective P2Y_6_ receptor antagonist MRS2578 (10 μM). N = 5; * denotes p<0.05 compared to vehicle control and MRS2578 (10 μM). (B) The detection of unmodified Rac1 is not affected by the selective P2Y_6_ receptor antagonist MRS2578 (10 μM). TcdA/B-induced Rac1 glucosylation and subsequent loss of a detectable Rac1 band is illustrated in vehicle- or MRS2578-treated Caco-2 cells at various time-points over 16 hrs. The western blot pictured is representative of 4 separate experiments.

We next sought to determine whether MRS2578 was directly inhibiting the function of TcdA/B, thus reducing CXCL8/IL-8 production. Pretreating cells with MRS2578 (10 μM) had no effect on the TcdA/B-induced cell death, as assessed by an LDH assay (Figure 3A) and failed to alter the loss of band signal in western blots targeting non-modified Rac1 (Figure 3B), an approach described previously by Genth et al. (2006) to assess TcdB-induced Rac1 modification [42]. Taken together these data suggest that MRS2578 inhibits CXCL8/IL-8 production and secretion through blockade of the P2Y_6_ receptor and not by inhibiting toxin function.

### P2Y_6_ receptor-inhibition attenuates TcdA/B-induced NFκB activity

**Figure 4:**
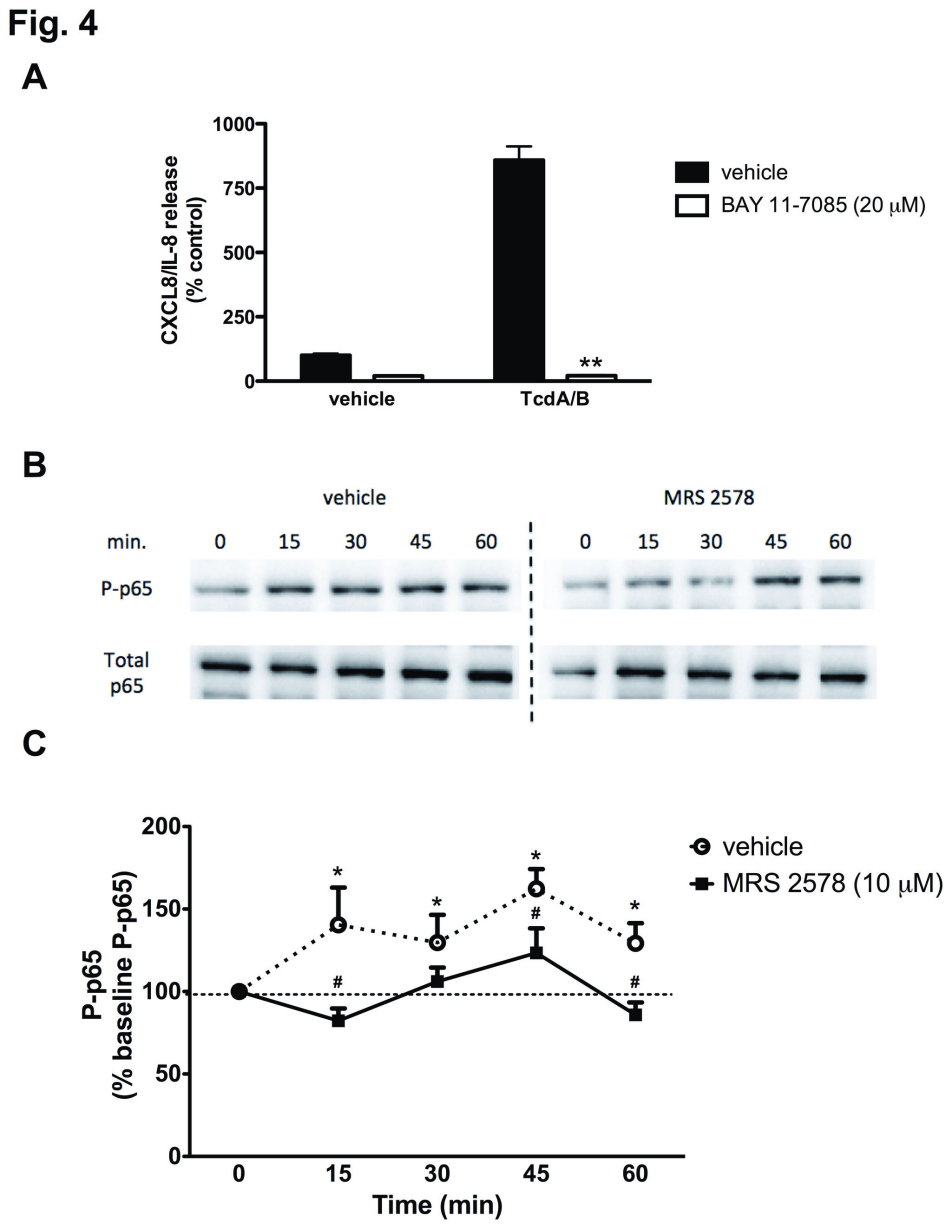
TcdA/B-induced CXCL8/IL-8 production from Caco-2 IECs involves the NFκB activation, an effect that is inhibited by pharmacological blockade of the P2Y_6_ receptor by MRS2578. (A) TcdA/B-induced CXCL8/IL-8 release is inhibited by pretreatment with the selective NFκB pathway inhibitor BAY 11-7085 (20 μM). N=6; ** denotes p<0.005 compared to vehicle-treated TcdA/B stimulated cells (10 μg/mL). (B) Representative western blot for phosphorylated p65 (P-p65) in lysates from TcdA/B (10 μg/mL) stimulated Caco-2 IECs over the course of 60 min in the presence of the P2Y_6_ antagonist MRS2578 (10 μM) or vehicle control (DMSO). (C) The summarized western blot data for P-p65 expressed as a percentage of the total p65. N = 4, *, denotes p<0.05 compared to time 0 min; # denotes p<0.05 compared to respective vehicle control (DMSO).

The production and release of CXCL8/IL-8 from IECs can be driven by the downstream activation of NFκB -dependent gene transcription [45]. Inhibition of NFκB signaling with BAY 11-7085 (20 μM) completely abolished TcdA/B-induced CXCL8/IL-8 production and release (Figure 4A). Since the P2Y_6_ antagonist MRS2578 reduced CXCL8/IL-8 responses to a similar degree as BAY 11-7085, we hypothesized that TcdA/B-induced P2Y_6_ receptor activation was activating NFκB -dependent CXCL8/IL-8 production. Interestingly, the P2Y_6_ receptor has been linked to NFκB signaling in a number of different cell types [37,38]. In Caco-2 cells, TcdA/B treatment led to a time-dependent increase in NFκB activity, as measured by western blotting for phosphorylated p65 (serine 536; P-p65; Figure 4B, summarized in Figure 4C). Blocking the P2Y_6_ receptor by pretreating the cells with MRS2578 (10 μM) significantly reduced TcdA/B-induced phosphorylation of p65 (Figure 4B, summarized in Figure 4C). The MRS2578-induced inhibition of p65 phosphorylation was pronounced in the first 30 min of TcdA/B treatment. Taken together, these data suggest that P2Y_6_-dependent activation of NFκB is driving TcdA/B-induced CXCL8/IL-8 production in Caco-2 IECs.

### TcdB triggers IL-8 release is abolished by apyrase and P2Y_6_ receptor blockade

**Figure 5:**
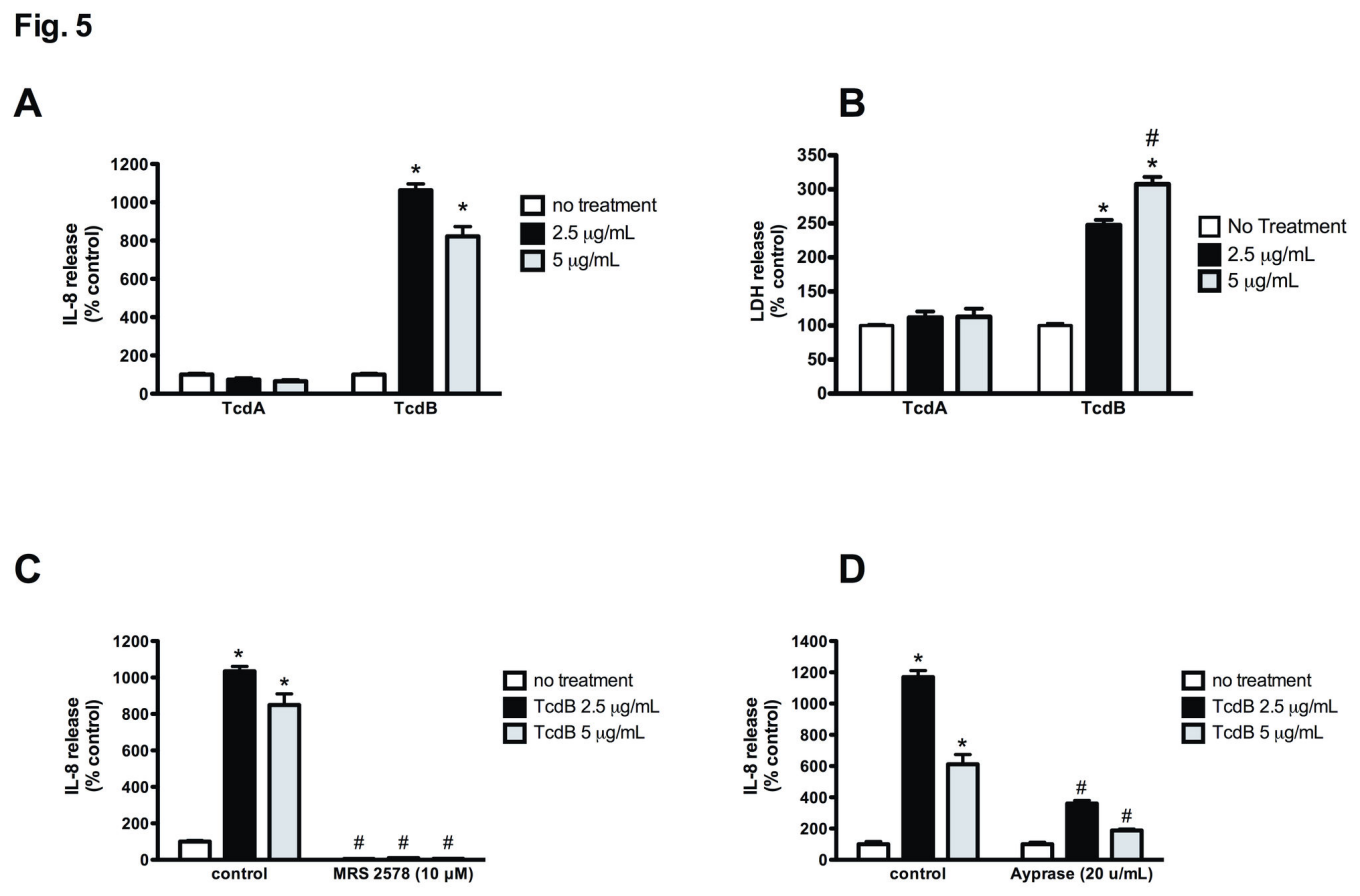
TcdB, but not TcdA triggers CXCL8/IL-8 production and release from Caco-2 cells in a manner dependent on extracellular nucleotides and the P2Y_6_ receptor. (A) CXCL8/IL-8 release from Caco-2 cells treated with purified TcdA or TcdB (16 hr). N = 5; * denotes p<0.05 compared to TcdA. (B) TcdB, but not TcdA, treatment of Caco-2 cells increases cell death as assessed by LDH release. N = 5; * denotes p<0.05 compared to TcdA. TcdB-induced CXCL8/IL-8 release is significantly reduced by (C) MRS2578 (10 μM) and (D) co-treatment with apyrase (20 u/mL). N = 5; * denotes p<0.05 compared to no treatment; # denotes p<0.05 compared to all groups.

To determine which component of our TcdA/B preparation was responsible for triggering the production and release of CXCL8/IL-8 from Caco-2 cells, we performed experiments using purified TcdA and TcdB. As reported previously, TcdB, but not TcdA, triggered the production of CXCL8/IL-8 from Caco-2 cells (Figure 5A) [7]. Similarly, only TcdB decreased cell viability as assess by an LDH assay (Figure 5B). As seen with TcdA/B, TcdB-induced CXCL8/IL-8 production and release was completely abolished following pretreatment with MRS2578 (10 μM, Figure 5C). Furthermore, co-treatment of Caco_2_ cells with apyrase significantly reduced TcdB-induced CXCL8/IL-8 release (Figure 5D). Together, these data suggest TcdB triggers CXCL8/IL-8 production through a nucleotide-dependent P2Y_6_-mediated mechanism. 

### P2Y_6_ receptor blockade attenuates TcdA/B-induced barrier dysfunction

**Figure 6:**
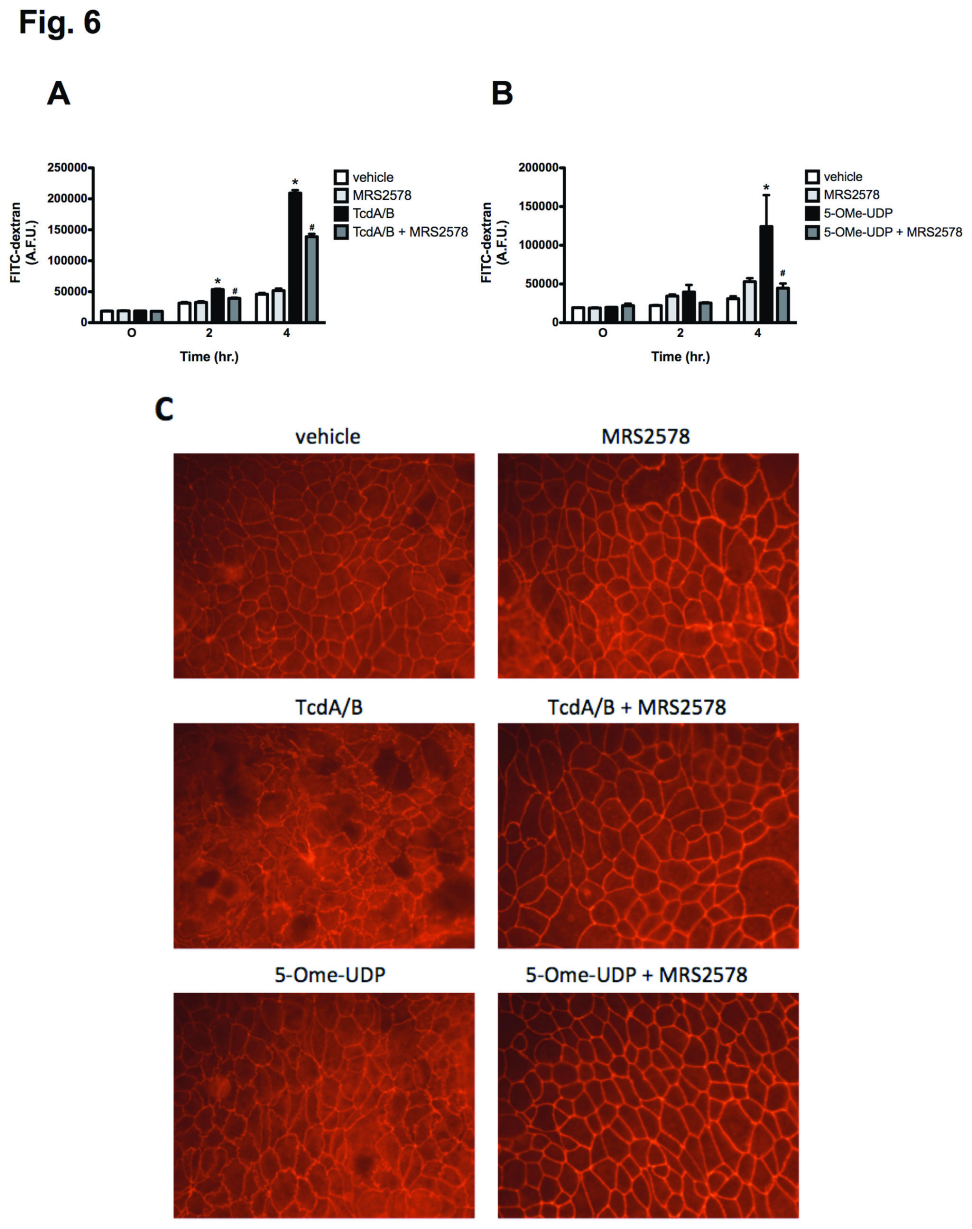
Inhibition of the P2Y_6_ receptor attenuates TcdA/B-induced intestinal epithelial barrier dysfunction in Caco-2 IECs. (A) TcdA/B-induced (10 μg/mL) FITC-flux is significantly reduced by the selective P2Y_6_ receptor antagonist MRS 2578 (10 μM). N=4; * denotes p<0.05 compared to vehicle and MRS2578. # denotes p<0.05 compared to TcdA/B. (B) 5-OMe-UDP (100 μM) increases FITC-flux in Caco-2 monolayers, an effect that is significantly attenuated by MRS2578 (10 μM). N=4; * denotes p<0.05 compared to vehicle and MRS2578. # denotes p<0.05 compared to 5-OMe-UDP. (C) Apical administration of TcdA/B (10 μg/mL) or 5-OMe-UDP (100 μM) for 4 hr triggers a redistribution of ZO-1 in Caco-2 monolayers, an effect that is blocked by pretreatment with MRS 2578 (10 μM; N=4).

In addition to triggering the release of inflammatory mediators such as CXCL8/IL-8, TcdA and TcdB can act directly on IECs to inhibit monomeric G-proteins leading to alterations in cytoskeletal function, reducing tight junction integrity, and ultimately increasing intestinal epithelial permeability. Interestingly, P2Y_6_ receptor activation has been associated with cytoskeletal remodeling in endothelial cells and alterations in vascular permeability [48]. Thus we examined whether the activation of the P2Y_6_ receptor might contribute to TcdA/B-induced intestinal epithelial barrier dysfunction. As we have reported previously [43], TcdA/B treatment led to an increase in FITC flux across the Caco-2 monolayer at 2 and 4 hrs post-treatment, indicating a loss of barrier function (Figure 6A). Interestingly, blocking the P2Y_6_ receptor with MRS2578 (10 μM) significantly attenuated this barrier defect at both time-points (Figure 6A). To explore further the role of the P2Y_6_ receptor in the changes in the barrier function observed, we treated Caco-2 monolayers with the potent P2Y_6_ receptor agonist 5-OMe-UDP in the presence and absence of MRS2578 (10 μM). Activation of the P2Y_6_ receptor with 5-OMe-UDP increased the flux of FITC across the cells in a fashion similar to TcdA/B peaking at 4 hr post-treatment. Furthermore, 5-OMe-UDP-induced disruption of the epithelial barrier was completely blocked by pretreating the monolayers with MRS2578 (Figure 6B). Of note, although P2Y_6_ receptor blockade significantly reduced TcdA/B-induced barrier dysfunction, this effect was not completely blocked, as was the case when cells were treated with 5-OMe-UDP. These data suggest that additional mechanisms are contributing to TcdA/B-induced barrier dysfunction. To visually assess the integrity of the cell-cell contacts responsible for the formation of a tight IEC monolayer, we performed immunofluorescence microscopy to examine the localization of zona occludens 1 (ZO-1), a key component of the tight junction complex. Caco-2 monolayers treated with TcdA/B or 5-OMe-UDP for 4 hr exhibited ZO-1 reorganization at the cell-cell contacts (Figure 6C). This response was prevented when the P2Y_6_ receptor was blocked by MRS2578 (10 μM). Taken together these data suggest that activation of the P2Y_6_ receptor can trigger increased permeability in monolayers through alteration of tight junction function. Furthermore, the ability of P2Y_6_ receptor blockade to protect Caco-2 monolayers from TcdA/B-induced barrier dysfunction suggests that the activation of the P2Y_6_ receptor during infection or inflammatory episodes may contribute to increased intestinal permeability, an effect that may worsen clinical outcomes in either case. 

### P2Y_6_ receptor blockade attenuates TcdA/B-induced intestinal inflammation and epithelial barrier dysfunction *in vivo*

**Figure 7:**
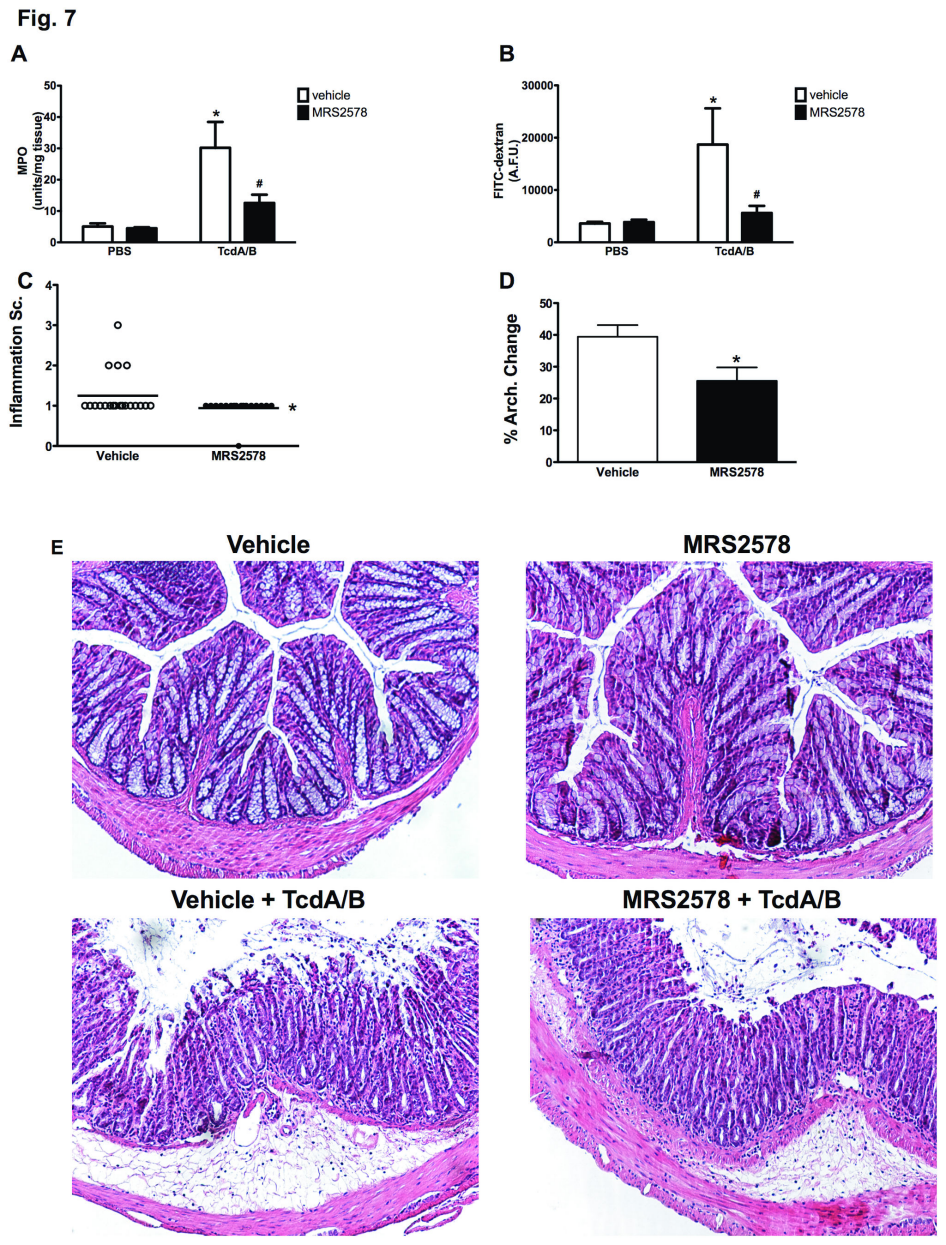
TcdA/B-induced intestinal inflammation and permeability are attenuated by inhibiting the P2y_6_ receptor *in* *vivo*. (A) Intrarectal instillation of TcdA/B (50 μg/100 μL for 4 hrs) triggers a significant increase in colonic tissue myeloperoxidase (MPO), an effect that is significantly reduced by pretreating mice with the P2Y_6_ inhibitor MRS 2578 (100 μL of 10 μM; in PBS via intrarectal instillation). N = 6/group; * denotes p<0.05 compared to PBS vehicle groups; # denotes p<0.05 compared to TcdA/B treatment with vehicle (DMSO). (B) Pretreating mice with MRS 2578 (100 μL of 10 μM; in PBS via intrarectal instillation) inhibits TcdA/B-induced increases in permeability as assessed by FITC-flux from the colonic lumen in to the serum. N = 6/group; * denotes p<0.05 compared to PBS vehicle groups; # denotes p<0.05 compared to TcdA/B treatment with vehicle (DMSO). (C) Pretreating mice with MRS 2578 reduces the histological inflammatory score and the (D) percentage of the colonic tissue section exhibiting architectural changes (% architecture change). N = 12 sections/group; * denotes P<0.05 compared to vehicle pretreatment. (E) Representative colonic sections stained with hematoxylin and eosin from mice treated with vehicle, MRS2578 alone, vehicle + TcdA/B and MRS2578 + TcdA/B; N = 6/group.

The pathogenesis of CDI is driven by the activity of TcdA and TcdB. *In vivo*, these toxins trigger the production of inflammatory mediators and cause extensive epithelial damage [49]. These events trigger an influx of inflammatory cells, primarily neutrophils and macrophages that may contribute to further tissue damage. Given our data suggesting that the P2Y_6_ receptor can mediate both the inflammation and barrier dysfunction associated with TcdA/B exposure, we sought to examine whether blocking the P2Y_6_ receptor could reduce TcdA/B-induced intestinal inflammation and permeability in a mouse model of toxin-induced inflammation and intestinal tissue damage [21]. Intrarectal instillation of TcdA/B increased intestinal inflammation and permeability, as indicated by increased colonic tissue MPO (an index of granulocyte infiltration; Figure 7A) and FITC flux from the gastrointestinal tract into the serum (an index of intestinal permeability; Figure 7B). Blocking the P2Y_6_ receptor by pretreating with MRS2578 (10 μM) attenuated both the inflammatory response (reduced colonic tissue MPO; Figure 7A) and intestinal barrier dysfunction (reduced FITC flux; Figure 7B). These trends were also reflected in our histological assessments. Pretreating mice with MRS2578 significantly reduced the inflammatory score (Figure 7C; representative sections in Figure 7E) and the percentage of the colonic tissue section exhibiting architectural changes (% architecture change; Figure 7D; representative sections in Figure 7E).

## Discussion

In the present study we outline a novel role for the P2Y_6_ receptor in mediating the production of CXCL8/IL-8 and triggering barrier dysfunction in Caco-2 cells exposed to *C. difficile* toxins. Pharmacological inhibition of the P2Y_6_ receptor attenuated TcdA/B-induced CXCL8/IL-8 production from Caco-2 cells and significantly reduced the inflammatory response in our *in vivo* model. Furthermore, TcdA/B-induced barrier dysfunction was attenuated by P2Y_6_ receptor blockade in both our *in vitro* and *in vivo* studies.

The immunostimulatory properties of *C. difficile* toxins TcdA and TcdB have been well characterized using *in vitro* and *in vivo* systems [49]. These toxins alter mitochondrial function [22,50,51], disrupt the cytoskeleton and cell-cell contacts in IECs [52,53] and trigger the production of inflammatory cytokines, such as IL-1β and CXCL8/IL-8, from various cell types [6-8]. Studies characterizing the induction of cytokine production have implicated the inflammasome and MAP kinase signaling pathways in these responses; however, the exact receptor(s) mediating these effects have yet to be identified [14,46,54-57]. 

In contrast to a direct method of triggering cytokine production, our data suggest that TcdA/B-induced cell stress and/or cell death drives the release of CXCL8/IL-8 through a paracrine pathway involving a P2Y_6_- and nucleotide-dependent mechanism. TcdA/B-treated Caco-2 cells released significant CXCL8/IL-8, which was associated with the accumulation of UDP in the culture supernatant and completely blocked by MRS2578, a selective P2Y_6_ receptor antagonist. This inhibitor had no effect on TcdA/B-induced cell death or Rac1 modification, suggesting its effect on CXCL8/IL-8 production was through the inhibition of the P2Y_6_ receptor. This notion is further supported by the observation that the CXCL8/IL-8 release and barrier dysfunction triggered by 5-OMe-UDP, a potent and selective P2Y_6_ receptor agonist, was completely blocked by MRS2578. 

When assessing the effects of the purified toxins on the induction of CXCL8/IL-8 production, we observed that only TcdB could trigger this response, an effect that was completely inhibited by blocking the P2Y_6_ receptor or co-treating with apyrase to breakdown extracellular nucleotides. Furthermore, only TcdB proved cytotoxic to Caco-2 cells suggesting that toxin-induced cell death and concurrent nucleotide release may be driving P2Y_6_ receptor-dependent CXCL8/IL-8 production and release in a paracrine fashion. Interestingly, the *C. difficile* toxin glucosyltransferase domain utilizes UDP-glucose as a substrate for the modification of monomeric G-proteins liberating UDP within the cell [58], hinting at an additional mechanism through which this nucleotide may accumulate in an intoxicated cell and enhance P2Y_6_ receptor signaling in adjacent cells upon its release. In contrast to our findings, Warny et al. (2001) reported that TcdA could trigger CXCL8/IL-8 production from monocytes, but this response did not involve extracellular nucleotides, as it was insensitive to apyrase [59]. These findings, along with our previous work demonstrating that TcdA and TcdB can trigger similar IL-1β responses in monocytes [14], suggest that *C. difficile* toxins elicit distinct cell-specific responses.

The P2Y_6_ receptor, coupled to Gq/11, can initiate a number of intracellular signaling events including intracellular calcium release via the production of IP_3_ and activation of various kinase pathways (e.g. protein kinase C, ERK1/2 and ROCK). The P2Y_6_ receptor has been previously linked to production of CXCL8/IL-8, evoked by UDP and other inflammatory stimuli [31,59,60], and may play a role in the induction of neutrophil migration [61]. Both AP-1- and NFκB-dependent CXCL8/IL-8 production have been associated with activation of the P2Y_6_ receptor, the former requiring intermediate ERK1/2 activation [31,37,38]. In our study, TcdA/B-induced CXCL8/IL-8 release from Caco-2 cells was completely abolished following pharmacological inhibition of the NFκB pathway, an effect mimicked by P2Y_6_ receptor blockade. Furthermore, we showed that P2Y_6_ receptor inhibition blocked toxin-induced activation of NFκB. 

In addition to mediating the production of CXCL8/IL-8, our studies support a role for the P2Y_6_ in the regulation of tight junctions during toxin exposure. Inhibiting the P2Y_6_ receptor attenuated TcdA/B-induced barrier dysfunction, as measured by FITC-dextran flux through a polarized Caco-2 monolayer. Furthermore, selectively activating the P2Y_6_ receptor with 5-OMe-UDP increased permeability, an effect that was completely blocked by MRS2578. The P2Y_6_ receptor has been implicated in regulating ion transport in epithelial cells [62-64], to our knowledge this is the first report outlining its role in regulating epithelial barrier function. Activation of the P2Y_6_ receptor in endothelial cells has been linked to vascular permeability [48]; however, this has yet to be reported in other cell types. Given its ability to activate ROCK and modify cytoskeletal function, it is plausible that P2Y_6_ receptor activation may alter cell-cell interactions in IEC monolayers. Interestingly, the permeability defects we observed in our studies were associated with a redistribution of ZO-1, a component of the epithelial tight junction complex, suggesting that P2Y_6_ receptor activation contributes TcdA/B-induced disruption of cell-cell contacts in Caco-2 monolayers. Despite the significant reduction in FITC-flux observed in TcdA/B-treated monolayers pretreated with MRS2578, this inhibitor did not completely abolish the barrier disruption, as was observed in 5-OMe-UDP treated cells, suggesting that additional mechanisms are contributing to TcdA/B-induced barrier dysfunction.

To translate our *in vitro* findings into the *in vivo* setting, we performed experiments in mice using our intrarectal toxin exposure model [21]. Previous studies assessing the functions of *C. difficile* toxins have injected TcdA or TcdB into isolated intestinal segments generated during a laparotomy [15,39,65,66]. Recently we’ve developed a non-invasive mouse model where we administer TcdA and/or TcdB via the intrarectal route and assess various parameters of inflammation and tissue damage in the colon [21]. Using this model we have shown previously that TcdA and TcdB synergize to trigger increased intestinal permeability and colonic tissue inflammation [21]. In the current study, pretreating mice with MRS2578, a P2Y_6_ antagonist, completely blocked TcdA/B-induced inflammation (as assessed by colonic MPO quantification) and intestinal barrier dysfunction (as assessed by flux of intestinally administered FITC-dextran into the serum). 

Although our data suggest that targeting the P2Y_6_ receptor may prove effective in the treatment of CDI, it is important to note that its ubiquitous expression will require targeted delivery of any therapeutic agent into the gastrointestinal tract with little systemic absorption. Furthermore, the P2Y_6_ receptor has been reported to play a role in the migratory capacity and/or function of various immune cells, including neutrophils [61,67] and macrophages, [68,69], both of which afford host defense during infection. As mentioned previously, some investigators have reported that mice lacking a functional immune capacity fail to resolve CDI in the absence of therapeutic intervention (i.e. without treatment with antibiotics that target *C. difficile*) [10-12]. Nevertheless, in concert with therapies designed to eradicate *C. difficile*, agents that selectively target intestinal P2Y_6_ signaling may prove useful in the treatment of CDI, especially in severe cases that exhibit an exaggerated immune response. 
